# Four-Year Outcomes of One-Anastomosis Gastric Bypass in Children and Adolescents with Obesity: Safety, Effectiveness, and Resolution of Obesity-Related Medical Conditions

**DOI:** 10.1007/s11695-025-07976-5

**Published:** 2025-07-08

**Authors:** Mohamad Hayssam ElFawal, Osama Taha, Mahmoud Abdelaal, Huneida Hamzeh, Zahi Hamdan, Dyaa Mohamad, Kareem El-Ansari, Hani Tamim, Walid El Ansari

**Affiliations:** 1 Beirut Arab University, Beirut, Lebanon; 2 Assiut University, Assiut, Egypt; 3 University of Balamand, Tripoli, Lebanon; 4 American Academy of Cosmetic Surgery Hospital, Dubai, United Arab Emirates; 5 St. George’s University, St. George’s, Grenada; 6 American University of Beirut, Beirut, Lebanon; 7 Ajman University, Ajman, United Arab Emirates

**Keywords:** Obesity, OAGB, Childhood, Adolescence, One anastomosis gastric bypass, Metabolic surgery, Bariatric surgery

## Abstract

**Background:**

Very few studies examined the safety and effectiveness of OAGB among adolescents. We undertook this task.

**Methods:**

Retrospective review of consecutive adolescents (*N* = 91, 11–21 years old) who underwent primary OAGB in Lebanon and Egypt (January 2013–January 2018). Data retrieved included anthropometric variables (weight, BMI, EWL%, TWL%), nutritional/metabolic outcomes (hemoglobin, protein, vitamin B12, albumin, Ca, HbA1c), and obesity-related conditions (T2DM, hypertension, depression, PCOS, OSA, and GERD). Data were retrieved preoperatively and at 1, 2, 3, and 4 years.

**Results:**

Mean age was 16.6 years, weight 117.6 kg, BMI 42 kg/m^2^, and 81.3% were females. By year 1, weight loss was large and significant, maintained through subsequent years (mean weight_year 4_ = 74.3 kg), mirrored by significant BMI reductions (mean BMI_year 4=_28.9 kg/m^2^). At year 1, mean EWL% was 80.2 ± 18.6% and TWL% 31.2 ± 5.8%, reaching 35.48 ± 8.85% and 91.26 ± 21.85% at year 4. At year four, HbA1c levels significantly decreased from preoperative 5.82 to 5.02%. Mean Hb, vitamin B12, albumin, protein, and calcium levels were significantly reduced from baseline to year 4, although the reduced levels remained within the normal reference ranges. All T2DM, PCOS, and hypertension cases achieved remission at year 1, maintained thereafter, with very few recurrences. OSA showed 83.3% resolution by year 1, with the rest achieving resolution at year 2. GERD was halved by year 1 and maintained. Complications were low (2.2%), with no mortality.

**Conclusion:**

OAGB is a safe and effective long-term procedure for adolescents. Weight loss and BMI reduction were significant, and remissions of obesity-related conditions were achieved and maintained. Larger studies are required.

## Introduction

Adolescent obesity and its associated medical conditions have long-term health impacts and comprise a serious public health concern worldwide [1–3]. Metabolic and bariatric surgery (MBS) is the most effective treatment modality for severe obesity [4], and among MBS procedures, one anastomosis gastric bypass (OAGB) is a frequently undertaken procedure [5], currently the third most common worldwide [6]. OAGB has been widely examined in adults [7] and has shown outstanding outcomes and low post-operative complication rates [8, 9]. However, data on its effectiveness and safety among children and adolescents remain scarce, resulting in a limited evidence base.

Indeed, only three studies have assessed the effectiveness of OAGB among adolescents [10–12]. These have shown that OAGB is effective and safe for this population group [10–12]. Notwithstanding, these studies displayed some limitations.

Certainly, the literature reveals knowledge gaps. Of these three studies, only one was dedicated to OAGB [11], while the other two examined OAGB as part of a comparison with sleeve gastrectomy [12], or with sleeve gastrectomy and Roux-en-Y gastric bypass [10]. In terms of sample sizes, two of these studies recruited modest numbers of participants, ranging from 22 [12] to 39 adolescents [11] and one assessed 84 adolescents [10], rendering the power of some of these studies to detect differences limited. As for outcomes, these studies focused narrowly on anthropometric outcomes, with no appraisals of nutritional or metabolic variables and their changes [10–12], despite the importance of such variables after MBS, particularly among adolescents who are in the development phase of life [2, 3, 13]. Pertaining to obesity-related medical conditions, two of the three studies did not examine any changes in obesity-related conditions [10, 12], despite that diabetes, hypertension, polycystic ovary syndrome, and other conditions can have a long-term impact on these young adults [14]. Other limitations include the short 1-year follow-up [12], and that these investigations were single-center, single-country studies [10–12].

Therefore, the current study aimed to bridge these knowledge gaps. We appraised the effectiveness and safety of OAGB at two centers in two countries, employing a larger series of 91 adolescents assessed at four time points after OAGB, appraising the changes across a range of anthropometric, nutritional, and metabolic outcomes and obesity-related medical conditions. The specific objectives were to assess, among these adolescent series, compared to their preoperative values, the post-OAGB changes at years 1, 2, 3, and 4, pertaining to (a) anthropometric variables [weight, body mass index (BMI), excess weight loss (EWL)%, total weight loss (TWL)%], (b) six nutritional and metabolic outcomes (hemoglobin, vitamin B12, protein, albumin, Ca, HbA1c), and (c) six obesity-related conditions [type 2 diabetes mellitus (T2DM), hypertension, depression, polycystic ovary syndrome (PCOS), obstructive sleep apnea (OSA), and gastro-esophageal reflux disease (GERD)]. In addition, the study sought to compare the changes for these variables at each time point with the previous time point in order to appraise year-on-year changes.

The findings of the current study would contribute to the currently limited evidence base of the effectiveness and safety of OAGB among adolescents and provide evidence for surgeons who are cautious to perform the procedure on this age group [10].

## Materials and Methods

### Study Design and Ethics

The study is a retrospective analysis of prospectively collected data of OAGB undertaken at one institution in Egypt and another in Lebanon during January 2013–January 2018. The study was approved by the Ethics Committees of both institutions, and no informed consent was required from patients. All measurement protocols and assessments were standardized across the two centers.

### Participants: Inclusion and Exclusion Criteria

We included all consecutive adolescent patients of both sexes, aged 11–21 years old with BMI > 40 kg/m^2^ or BMI > 35 kg/m^2^ and with medical problems who had undergone OAGB during the study period, in line with American Academy of Pediatrics [15] and IFSO guidelines [16]. Exclusion criteria were patients who had any previous MBS or those with genetic conditions such as Prader Willi and Down syndrome. A total of 91 adolescents were included in the current analysis.

### Surgical Technique

OAGB was undertaken using the laparoscopic approach, making a lesser curvature-based gastric pouch about 15–18 cm in length from the gastroesophageal junction over a 40 French oral tube using multiple firings of a 60-mm linear stapler. A 45-mm linear stapler was used for the gastro-jejunostomy, bypassing 150–180 cm of the small bowel from the ligament of Treitz as the biliopancreatic (afferent) limb in an antecolic fashion. The defect was closed with one or two layers of PDS 2–0 sutures, and no additional anti-reflux stitches were undertaken. The Peterson defect was not closed and a routine leak test with methylene blue was conducted. The surgeries were performed by the same experienced consultant surgeon at each institution.

### Preoperative Evaluation

This included history, physical examination, and other specialist consultations with other departments was required. The surgical details, risks, benefits, and long-term consequences of the OAGB were discussed in detail during the initial meeting of the patient and family with the surgeon and dietician. Laboratory investigations included complete blood count, glycated hemoglobin (HbA1c), prothrombin concentration, renal function tests, liver function tests, and thyroid function.

### Statistical Analysis

Statistical Package for Social Sciences (SPSS) version 28 was used for data management and analysis. Data were expressed as means with standard deviation and range for continuous variables or frequency and percent for categorical variables. Paired *t*-test was used to assess the association between values at baseline and each time point. A *P*-value of < 0.05 was used to indicate statistical significance. A mixed effects model assessed time effects. Stratification was undertaken by Centre and by age group. Missing data was not imputed. We enrolled all the consecutive patients at the two centers; hence, we did not undertake a formal power calculation for sample size.

### Criteria for Diagnosis and Remission of Associated Medical Conditions

Preoperative diagnosis of T2DM was based on the 2008 American Diabetes Association (ADA) criteria, after the addition of HbA1c by the International Expert Committee [17, 18]. T2DM remission was defined as HbA1c < 6.0% without anti-diabetic medication for 1 year, based on the ADA criteria [19], and in line with previous research [20–25].

Hypertension was defined as resting blood pressure ≥ 140/90 mmHg or if the patient was on antihypertensive medication/s. Hypertension remission was defined as no use of antihypertensive medications with systolic blood pressure ≤ 135 and diastolic blood pressure ≤ 85 mmHg [11]. Patients were considered to have OSA when they had a recorded diagnosis of OSA and were receiving treatment. OSA remission was considered when symptoms had been recorded as resolved and OSA treatment stopped [26]. Diagnosis and remission of PCOS were based on the 2011 Chinese diagnostic criteria (meeting any two of three conditions of androgen excess, ovulatory dysfunction, and polycystic ovaries) [27, 28]. Patients reporting reflux symptoms or taking daily medication for GERD were recorded as diagnosed with GERD [26]. Diagnosis and remission of depression were based on the psychiatric team’s diagnosis.

### Post-Operative Care

After surgery, patients are routinely followed up by a multi-disciplinary team of bariatric surgeons, dietitians, and physiotherapists. Follow-up visits are scheduled at 2 weeks and then monthly for a year, and yearly thereafter. Dietitians and physiotherapists individually counsel adolescents and their family members on the routine post-surgery dietary intake and physical activity in accordance with established international guidelines [29, 30].

### Data Collection

Data retrieved included pre- and post-operative anthropometric variables (weight, BMI, EWL%, TWL%), nutritional and metabolic outcomes (hemoglobin, hematocrit, vitamin B12, protein, albumin, Ca, HbA1c), and obesity-related conditions (T2DM, hypertension, depression, PCOS, OSA, and GERD). All data were retrieved pre-operatively and at the four time points under examination (years 1, 2, 3, and 4 after surgery).

## Results

### General Characteristics of the Sample

The series comprised 91 consecutive patients with a mean age of 16.6 years and 81.3% were females. Mean weight was 117.6 kg (range 89–160 kg), and mean BMI was 42 kg/m^2^ (Table 1).

**Table 1:** Preoperative characteristics of adolescents

Characteristic	Value
Sex	
Male	17 (18.7)
Female	74 (81.3)
Age (years)	
Mean ± SD	16.6 ± 1.46
Range	13–19
Weight (kg)	
Mean ± SD	117.61 ± 18.44
Range	89–160
BMI (kg/m^2^) mean ± SD	
Mean ± SD	42 ± 4.72
Range	34.3–55.5
Obesity-related medical conditions	
Type 2 diabetes	18 (20.0)
Hypertension	10 (11.0)
Depression	4 (4.4)
GERD	14 (15.4)
PCOS	20 (27.4)
OSA	18 (20.0)

All cell values represent frequency (%), unless otherwise stated. *SD*, standard deviation; *GERD*, gastroesophageal reflux disease; *PCOS*, polycystic ovary syndrome; *OSA*, obstructive sleep apnea

Hospital stay was one post-operative day for all cases. There were no early post-operative complications except in one patient who developed hemorrhage 8 hours post-operative and was managed conservatively. As for late complications, during the first year, one female patient presented with severe iron deficiency anemia requiring initial intravenous supplementation followed by oral supplementation. Both patients recovered. There was no mortality across the series.

### Anthropometric Changes

Table 2 depicts that there was a large and significant weight loss by year 1, followed by maintenance of the lost weight across the four time points, reaching a mean weight of 74.3 kg at year 4. This weight decrease was mirrored by significant reductions in BMI over time, reaching 28.9 kg/m^2^ at year 4. Hence, by year 1, mean EWL% was 80.2 ± 18.6% and TWL% was 31.2 ± 5.8%, reaching 35.48 ± 8.85% and 91.26 ± 21.85%, respectively, at year 4.

**Table 2:** Anthropometric changes among adolescents across four time points after OAGB (*N* = 91)

Characteristic	Pre-op	Year 1	***p***	Year 2	***p***	Year 3	***p***	Year 4	***p***^*a*^	***p***^*b*^
Weight (kg)	117.61 ± 18.44	80.88 ± 14.16	< *0.001*	76.36 ± 12.75	< *0.001*	74.32 ± 10.53	< *0.001*	74.27 ± 10.79	< *0.001*	< *0.001*
*n*	91	91		89		86		75		
BMI (kg/m^2^)	42.0 ± 4.72	28.87 ± 3.89	< *0.001*	27.25 ± 3.61	< *0.001*	26.63 ± 3.03	< *0.001*	26.59 ± 3.42	< *0.001*	< *0.001*
*n*	91	91		89		86		75		
EWL%	**—**	80.20 ± 18.59	**—**	89.63 ± 18.63	< *0.001*	91.91 ± 18.58	< *0.001*	91.26 ± 21.85	*0.004*	< *0.001*
*n*	91	91		89		86		75		
TWL%	**—**	31.20 ± 5.81	**—**	35.00 ± 7.29	< *0.001*	35.98 ± 7.29	< *0.001*	35.48 ± 8.85	< *0.001*	< *0.001*
*n*	91	91		89		86		75		

Cell values represent mean±standard deviation; *Pre-op* pre-operative; *BMI*, body mass index; *EWL%*, excess weight loss percentage; *TWL%*, total weight loss percentage; ^*a*^ values indicate comparisons of given year with preoperative value; ^*b*^* p* value for the mixed model, accounting for the longitudinal analysis over 4 years; italicized cells indicate statistical significance; **—**: not applicable; *n* number of patients with data at the given time point

Table 3 provides the details of the magnitude of anthropometric changes (∆) compared with preoperative values and with values of the previous time point. One year after surgery, there were significant mean changes (∆) in weight and BMI from preoperative values amounting to − 36.73 ± 9.10 kg and − 13.13 ± 2.98 kg/m^2^, respectively. For years 2 and 3, the initial weight and BMI reductions were maintained, with additional significant weight losses ranging between − 4.66 and − 1.32 kg, and BMI reductions ranging between − 1.64 and − 0.42 kg/m^2^. At year 4, the mean changes (∆) from preoperative values were significant, totaling − 41.96 ± 14.32 kg (weight) and − 14.89 ± 4.51 kg/m^2^ (BMI).

**Table 3:** Magnitude of anthropometric changes: comparisons with preoperative values and previous time points (*N* = 91)

Variable			Year 1*	Year 2	Year 3	Year 4
Weight (kg)	Timepoint vs pre-op	*∆*	− 36.73 ± 9.10	− 41.45 ± 10.71	− 42.71 ± 13.07	− 41.96 ± 14.32
		*P*	< *0.001*	< *0.001*	< *0.001*	< *0.001*
		*n*	91	89	86	75
	Timepoint vs previous one	*∆*	− 36.73 ± 9.10	− 4.66 ± 5.12	− 1.32 ± 4.73	0.54 ± 4.92
		*P*	< *0.001*	< *0.001*	*0.01*	0.35
		*n*	91	89	84	74
BMI (kg/m^2^)	Timepoint vs pre-op	*∆*	− 13.13 ± 2.98	− 14.77 ± 3.36	− 15.20 ± 4.06	− 14.89 ± 4.51
		*P*	< *0.001*	< *0.001*	< *0.001*	< *0.001*
		*n*	91	89	86	75
	Timepoint vs previous one	*∆*	− 13.13 ± 2.98	− 1.64 ± 1.81	− 0.42 ± 1.66	0.21 ± 1.79
		*P*	< *0.001*	< *0.001*	*0.02*	0.31
		*n*	91	89	84	74
EWL%	Timepoint vs pre-op	*∆*	**—**	9.49 ± 12.37	11.26 ± 18.69	8.98 ± 25.78
		*P*	**—**	< *0.001*	< *0.001*	*0.004*
		*n*	91	89	86	75
	Timepoint vs previous one	*∆*	**—**	9.49 ± 12.37	1.75 ± 10.32	− 1.95 ± 12.44
		*P*	**—**	< *0.001*	0.13	0.18
		*n*	**—**	89	84	74
TWL%	Timepoint vs pre-op	*∆*	**—**	3.82 ± 4.41	4.69 ± 6.88	3.92 ± 4.41
		*P*	**—**	< *0.001*	< *0.001*	< *0.001*
		*n*	91	89	86	75
	Timepoint vs previous one	*∆*	**—**	3.82 ± 4.41	0.87 ± 3.93	− 0.58 ± 4.41
		*P*	**—**	< *0.001*	*0.045*	0.26
		*n*	**—**	89	84	74
Prevalence of weight regain (≥ 10 kg) ^*a*^	Timepoint vs pre-op	*N (%)*	0 (0.0)	0 (0.0)	0 (0.0)	0 (0.0)
		*P*	**—**	**—**	**—**	**—**
		*n*	91	89	86	75
	Timepoint vs previous one	*N %*	0 (0.0)	2 (2.2%)	0 (0.0)	3 (4.1%)
		*P*	**—**	UTC	**—**	UTC
		*n*	91	89	84	74

*Pre-op*, pre-operative; *BMI*, body mass index, *EWL%*, excess weight loss percentage; *TWL%*, total weight loss percentage; **—**, not applicable; *UTC*, unable to compute; italicized cells indicate statistical significance; *n* number of patients with data at the given time point

At year 2, the mean change compared to year 1 in EWL% and TWL% was 9.49 ± 12.37% and 3.82 ± 4.41%, respectively, which were maintained for subsequent years. At year 4, mean changes (∆) from preoperative value were significant, amounting to 8.98 ± 25.78% (EWL%) and 3.92 ± 4.41% (TWL%). There was no significant weight regain across the series during the duration of the study.

### Metabolic and Nutritional Changes

Table 4 shows the changes across four time points after OAGB. At year 4, HbA1c levels significantly decreased from a preoperative 5.82 to 5.02%. Similarly, the mean Hb level was significantly reduced from 12.8 ± 1.5_preop_ to 11.7 ± 1.5_year 4_, and mean vitamin B12 was significantly reduced from 454.4 ± 168.3_preop_ to 411 ± 190.6_year 4_. Serum albumin level was significantly decreased from 4.3 ± 0.3 g/dL to 3.9 ± 0.2 g/dL. Similarly, significant reductions across time were also observed for protein and calcium. Despite the reductions, the reduced levels of all the metabolic and nutritional variables under examination were still within the normal reference ranges.

**Table 4:** Metabolic and nutritional changes among adolescents across four time points after OAGB (*N* = 91)

Characteristic	Pre-op	Year 1	*p*	Year 2	*p*	Year 3	*p*	Year 4	*p*
Hb (gm/dL)	12.82 ± 1.46	12.03 ± 1.69	< *0.001*	11.88 ± 1.55	< *0.001*	11.85 ± 1.50	< *0.001*	11.71 ± 1.46	< *0.001*
*n*	89	87		84		64		43	
Vitamin B12 (pg/mL)	454.4 ± 168.3	419.4 ± 176.6	*0.001*	399.6 ± 152.3	< *0.001*	387.1 ± 174.7	< *0.001*	411.0 ± 190.6	0.067
*n*	62	69		63		61		52	
Protein (g/dL)	7.35 ± 0.44	6.88 ± 0.42	< *0.001*	6.85 ± 0.48	< *0.001*	6.88 ± 0.53	< *0.001*	6.98 ± 0.55	*0.001*
*n*	67	67		67		53		39	
Albumin (g/dL)	4.29 ± 0.33	4.07 ± 0.33	< *0.001*	4.00 ± 0.25	< *0.001*	4.04 ± 0.27	< *0.001*	3.89 ± 0.21	< *0.001*
*n*	57	61		62		52		47	
Calcium (mg/dl)	8.92 ± 0.76	8.60 ± 0.67	< *0.001*	8.74 ± 0.76	0.12	0.63 ± 0.96	0.15	8.63 ± 0.76	*0.01*
*n*	31	31		31		24		19	
HbA1c (%)	5.82 ± 0.93	5.01 ± 0.41	< *0.001*	4.86 ± 0.30	< *0.001*	4.98 ± 0.33	< *0.001*	5.02 ± 0.45	< *0.001*
*n*	63	56		50		47		37	

Cell values represent ± mean standard deviation, *Pre-op* pre-operative, *Hb* hemoglobin, *HbA1C* glycated hemoglobin, *p* values indicate comparisons of the value of the given year with preoperative value, italicized cells indicate statistical significance, *n* number of patients with data at the given time point

### Changes in Obesity-Related Medical Conditions

Table 5 depicts that all patients with preoperative T2DM (18/18), and almost all patients with PCOS (19/20) and hypertension (9/10) achieved remission at year 1 that was maintained thereafter through the study period, with very few recurrences (one patient with T2DM recurrence at year 4, four cases with PCOS recurrence at year 3, and one patient with persistent hypertension throughout the study period). OSA was considerably reduced (83.3% resolution) by year 1, with the few remaining cases achieving resolution at year 2 and no OSA across the sample by year 4. GERD decreased from 15.4% at baseline to 8.2% at year 1, with no new cases thereafter. As for depression, three of the four patients remained the same, while the fourth worsened, had two suicide attempts, and extensive medical therapy was provided.

**Table 5:** Changes in associated medical conditions among adolescents at four time points after OAGB (*N* = 91)

Condition	Pre-op	Year 1	*p*	Year 2	*p*	Year 3	*p*	Year 4	*p*
Diabetes	18 (20.0)	0 (0.0)	< *0.001*	0 (0.0)	< *0.001*	0 (0.0)	< *0.001*	1 (1.4)	< *0.001*
*n*	90	86		79		77		70	
PCO	20 (27.4)	1 (1.4)	< *0.001*	0 (0.0)	< *0.001*	3 (4.3)	< *0.001*	3 (5.2)	*0.001*
*n*	73	73		73		69		58	
Hypertension	10 (11.0)	1 (1.1)	*0.004*	1 (1.1)	*0.004*	1 (1.2)	*0.008*	1 (1.3)	*0.008*
*n*	91	91		91		86		76	
OSA	18 (20.0)	3 (3.3)	< *0.001*	1 (1.1)	< *0.001*	1 (1.1)	< *0.001*	0 (0.0)	< *0.001*
*n*	90	91		91		87		80	
GERD	14 (15.4)	7 (8.2)	0.18	7 (8.1)	0.18	6 (7.1)	0.08	6 (8.0)	0.12
*n*	91	85		86		85		75	
Depression	4 (4.4)	4 (4.4)	1.0	4 (4.4)	1.0	4 (4.9)	1.0	4 (6.1)	1.0
*n*	91	91		91		81		66	

Comparison is for given time compared to perioperative value; cell values represent frequency (percent); *n* denominator, *Pre-op* pre-operative, *HTN* hypertension, *GERD* gastroesophageal reflux disease, *PCOS* polycystic ovary syndrome, *OSA* obstructive sleep apnea, italicized cells indicate statistical significance, *n* number of patients with data at the given time point

### Associations of Centre, Patient Age, and Loss to Follow-Up with Outcomes

In terms of Centre, for the first 2 years, both the centers in Egypt and Lebanon were similar in their observed weight, BMI, EWL%, and TWL reductions; however, for years 3 and 4, Centre 2 exhibited significantly more weight, BMI, and EWL% reductions (Table 6). Age exhibited no associations with any anthropometric outcome across all time points (Table 7). Pertaining to loss to follow-up, although our patients who were lost to follow-up (LTFU) were not different compared to those who completed the study across all the variables under examination, they tended to have heavier BMI, from Centre 2, and have lower Ca levels (Table 8).

**Table 6:** Anthropometric changes among adolescents by Centre across four time points after OAGB

Characteristic	Pre-op	Year 1	Year 2	Year 3	Year 4
Weight (kg)					
Centre 1	120.91 ± 21.84	83.22 ± 16.84	78.53 ± 15.68	77.73 ± 12.97	78.46 ± 13.66
* n*	34	34	34	30	24
Centre 2	115.65 ± 15.94	79.49 ± 12.24	75.02 ± 10.48	72.50 ± 8.54	72.29 ± 8.60
* n*	57	57	55	56	51
* P*	0.11	0.11	0.13	*0.01*	*0.01*
BMI (kg/m^2^)					
Centre 1	43.04 ± 5.53	29.62 ± 4.65	29.98 ± 4.60	27.83 ± 3.39	28.02 ± 3.89
* n*	34	34	34	30	24
Centre 2	41.38 ± 4.10	28.42 ± 3.31	26.80 ± 2.80	25.98 ± 2.62	25.92 ± 2.98
* n*	57	57	55	56	51
* P*	0.13	0.15	0.18	*0.01*	*0.03*
EWL%					
Centre 1	**—**	77.74 ± 19.69	87.48 ± 21.68	86.19 ± 19.44	84.82 ± 22.56
* n*	34	34	34	30	24
Centre 2	**—**	81.62 ± 18.06	90.96 ± 16.54	94.97 ± 17.52	94.29 ± 21.05
* n*	57	57	55	56	51
* P*	**—**	0.17	0.20	*0.02*	*0.04*
TWL%					
Centre 1	**—**	31.11 ± 6.51	34.93 ± 6.75	34.52 ± 7.09	33.52 ± 8.33
* n*	34	34	34	30	24
Centre 2	**—**	31.23 ± 5.37	35.04 ± 5.80	36.77 ± 7.33	36.39 ± 9.02
* n*	57	57	55	56	51
* P*	**—**	0.91	0.94	0.18	0.19

Cell values represent mean ± standard deviation; *Pre-op*, pre-operative; *BMI*, body mass index; *EWL%*, excess weight loss percentage; *TWL%*, total weight loss percentage; *p* values indicate comparisons across centers for the given year; italicized cells indicate statistical significance; **—**, not applicable; *n* number of patients with data at the given time point; *Centre 1* Lebanon; *Centre 2* Egypt

**Table 7:** Anthropometric changes among adolescents by age group across four time points after OAGB

Characteristic	Pre-op	Year 1	Year 2	Year 3	Year 4
Weight (kg)					
11–14 y	112.0 ± 12.23	77.86 ± 7.99	76.00 ± 8.76	74.57 ± 7.70	74.57 ± 10.37
* n*	7	7	7	7	7
15–17 y	115.62 ± 16.10	78.94 ± 13.12	74.72 ± 12.11	72.56 ± 9.08	73.05 ± 9.89
* n*	52	52	50	48	44
18–21 y	122.50 ± 22.15	84.70 ± 16.21	79.00 ± 14.25	77.00 ± 12.68	76.42 ± 12.48
* n*	32	32	32	31	24
* P*	0.16	0.16	0.34	0.19	0.47
BMI (kg/m^2^)					
11–14 y	42.41 ± 4.82	29.52 ± 3.66	28.82 ± 3.85	28.27 ± 3.39	28.29 ± 4.51
* n*	7	7	7	7	7
15–17 y	41.55 ± 4.76	28.42 ± 4.12	26.88 ± 3.94	26.15 ± 2.97	26.21 ± 3.41
* n*	52	52	50	48	44
18–21 y	42.63 ± 4.71	29.45 ± 3.55	27.49 ± 2.97	26.99 ± 2.96	26.80 ± 3.05
* n*	32	32	32	31	24
* P*	0.58	0.45	0.38	0.16	0.31
EWL%					
11–14 y	**—**	76.88 ± 21.46	81.45 ± 22.17	83.58 ± 18.79	84.64 ± 24.90
* n*	7	7	7	7	7
15–17 y	**—**	82.69 ± 19.71	91.76 ± 20.67	94.22 ± 20.74	92.61 ± 25.08
* n*	52	52	50	48	44
18–21 y	**—**	76.87 ± 16.25	88.09 ± 13.76	90.18 ± 14.34	90.72 ± 13.44
* n*	32	32	32	31	24
* P*	**—**	0.34	0.33	0.30	0.67
TWL%					
11–14 y	**—**	30.21 ± 6.52	31.93 ± 6.40	33.06 ± 6.79	33.17 ± 8.12
* n*	7	7	7	7	7
15–17 y	**—**	31.57 ± 5.99	35.22 ± 6.85	36.17 ± 7.90	35.29 ± 9.84
* n*	52	52	50	48	44
18–21 y	**—**	30.78 ± 5.59	35.32 ± 4.75	36.34 ± 6.42	36.49 ± 7.15
* n*	32	32	32	31	24
* P*	**—**	0.74	0.39	0.55	0.67

Cell values represent mean ± standard deviation; *Pre-op*, pre-operative; *BMI*, body mass index; *EWL%*, excess weight loss percentage; *TWL%,* total weight loss percentage; *p* values indicate comparisons across age groups for the given year; italicized cells indicate statistical significance; **—**, not applicable; *n* number of patients with data at the given time point

## Discussion

IFSO position statements and other reports have documented that children with obesity are at elevated risk of developing physical and psychosocial obesity-related conditions, and in the absence of lifestyle changes, they have about 10–20 years reduction in life expectancy and can develop serious obesity-related medical conditions later in life [9, 31–33]. As non-operative therapy has poor outcomes in studies among adolescents [34, 35], MBS is an effective alternative to achieve healthy weight and avoid the medical consequences of obesity [36, 37].

A multi-society expert panel concluded that adolescent obesity requires more intensive and aggressive treatment compared to adult obesity [38]. Whilst sleeve gastrectomy (SG) and Roux-en-Y gastric bypass (RYGB) among adolescents have been shown to be safe and effective [12, 39, 40], studies of OAGB outcomes have mainly been among adults [9, 41–44]. With only three published studies on the effectiveness and safety of OAGB among adolescents, the evidence base remains very limited, with little guidance for practitioners.

The current study bridged this knowledge gap and appraised the effectiveness and safety of OAGB among a series of 91 adolescents at two centers in two countries, assessing a wide range of anthropometric, nutritional, and metabolic outcomes, as well as the resolution of six obesity-related conditions at four time points compared to baseline values.

Our main findings were that there was significant weight loss and anthropometric improvements by year 1 after OAGB that were maintained throughout the study period. This was accompanied by significant improvements in the six pre-operative obesity-associated conditions except for depression. Whilst we also observed significant and healthy reductions in HbA1c, there were nevertheless reductions in Hb, vitamin B12, protein, albumin, calcium, and levels across time, although the reduced levels of all the variables under examination were still within the normal reference ranges. Below, we detail each of these findings.

In terms of anthropometry, by year 1, the current series’ mean TWL% was 31.2%, comparable to findings from Iran (35.3%) [10] but less than those in Spain (42.3%) [11]. Similarly, our 1-year post-operative EWL% was 80.2%, reaching 91.26% at year 4, comparable to EWL% reported by others 5 years after OAGB (94.3%) [10, 45]. A study compared OAGB to SG and RYGB among adolescents and showed that at 1 year, OAGB achieved significantly higher TWL% and higher BMI loss compared to both procedures [10]. Likewise, research among children reported that the TWL% at year 1 was quite similar for SG (26%) and RYGB (28%) [46], both of which were lower than the 1-year findings (31.2%) in the current series. These findings suggest OAGB may offer greater weight loss than other MBS options in adolescents, an observation that could inform procedure selection for appropriate adolescent candidates. The OAGB procedure induces weight loss and metabolic changes through several physiological mechanisms (restriction, malabsorption, gut hormonal changes, dumping syndrome which discourages the intake of calorie-dense foods) [47].

As for remissions of obesity related medical condition rates at year 1, in the current series, these amounted to 100% for T2DM and 90% for hypertension, similar to other research [11]. In terms of GERD, we observed about 50% reduction (from 15.4% preop to 8% post-op, with no de novo cases), less that rates reported in the only study that assessed it, where pre-operative GERD symptoms (23.1%) completely disappeared after OAGB with no new onset cases [11]. GERD remission rates after OAGB among adults’ amount to 72.2% remission. Pertaining to OSA, remission in the current series was 83.3%, but as no previous study has evaluated OSA remission after OAGB among adolescents, our findings are comparable to OSA remission after OAGB in adults [48–50]. Regarding PCOS, few studies have investigated the effects of MBS on PCOS and no studies assessed the evolution of PCOS after OAGB among adolescents, despite the central role that obesity plays in the pathophysiology of PCOS [51]. In the present series, post-operative remission of PCOS was 95%, which was similar to rates reported among adults after SG and RYGB studies [50, 52, 53].

As for depression, its progression after MBS appears to be inconsistent, as some studies suggest that MBS alleviates depression in patients with obesity [54], while others invite caution about the increased risk of adverse mental health after MBS, such as suicide and self-harm [55]. Our findings agree with the latter, as for three of the four patients with depression, the condition remained the same, while the fourth worsened, had two suicide attempts, and required extensive medical therapy. Hence, we advocate the need for dedicated mental health support, as weight improvement alone might not improve mood among adolescents. Further studies are required to elucidate the complex relationships between MBS and depression.

Nutritional deficiencies remain a concern after MBS that have a malabsorptive component. The current series displayed significant reductions of Hb, albumin, protein, calcium, and HbA1c levels across time; however, the reduced levels of all these variables under examination were still within the normal ranges. The literature reveals inconsistent findings of nutritional effects after OAGB. For instance, a study of 39 adolescents after OAGB reported that there were no cases of malnutrition [11]. Another study reported a series of 26 adult patients who had undergone OAGB with biliopancreatic limb > 200 cm (mean length 320 cm) however, due severe malnutrition, underwent reversal of OAGB to normal anatomy [56]. In our series, the biliopancreatic limb was 150–180 cm, which might explain why the reductions of nutritional variables, although significant, were not of large magnitude and were not reduced below the normal reference ranges. As for HbA1c, in the current series, it was reduced from 5.8 to 4.9% at year 2 after OAGB, concurring with findings of OAGB research among adults where mean HbA1c decreased from 9.6 ± 1.3 to 5.7 ± 1.5% at the 12-month follow-up to reach 5.8 ± 0.9% at the 3-year follow-up [43]. Such HbA1c findings are superior to those of medically treated patients where HbA1c worsened from 6.4 to 7.8% at year 2, but improved from 6.8 to 5.5% over the same time period among patients who underwent MBS [57].

The current study observed no age effects for any of the anthropometric outcomes across all time points, suggesting that the effectiveness of OAGB is uniform across various adolescent age groups, and that “younger” adolescents will benefit as much as “older” adolescents. However, there were mild Centre effects evident for years 3 and 4, where one Centre exhibited significantly more weight, BMI, and EWL% reductions. It is difficult to speculate the reasons behind such mild Centre effects.

As for LTFU, recent reports have uncovered important features pertaining to this group of patients [58]. Based on the values of the primary outcome of the current study (anthropometric values) (Table 2), our LTFU rates were 0%_year 1_ (91/91), 2.2%_year 2_ (2/91), 5.5%_year 3_ (5/91), and 17.5%_year 4_ (16/91). These rates are all below the universal (acceptable) 20% LTFU rate [58]. We are unable to compare our LTFU rates with others as adolescent OAGB studies seem to have altogether excluded those LTFU from the study, despite that the studies appraised several time points: in Spain, a 5-year follow-up of children and adolescent OAGB at a European IFSO Excellence Center excluded those LTFU [11], and in Iran, a 5-year cohort study on the safety and efficacy of OAGB in children and adolescents excluded those LTFU [10].

In addition, the preoperative characteristics of those LTFU were not different compared to those who completed the study across all the variables under examination except for three variables. A further interesting observation is that our non-concordance increased with age, as our LTFU steadily increased with age, starting at 0% for the 11–14-year-old age group, increasing to 18.2% for the 15–17-year-old, and reaching a LTFU rate of 33% for the 18–21-year-old age group (Table 8). This could be due to the fact that younger adolescents are generally more bonded with their parents and families who might be involved in the follow-ups, while older adolescents could be more independent. This suggests that older adolescents might require a “watchful eye” in monitoring their follow-ups, as well as direct psychological support as to the importance of such follow-ups.

**Table 8:** Comparison of pre-operative values of patients who completed the study and those lost to follow-up at year 4

**Variable**	**Patients** (*N* = 91)		***P***
	Not LTFU	LTFU	
	*n* = 75	*n* = 16	
Demography			
Gender			0.73
Male	15 (20.0)	2 (12.5)	
Female	60 (80.0)	14 (87.5)	
Age, years (M ± SD)	16.55 ± 1.49	17.13 ± 1.20	0.15
Age Bracket (years)			0.31
11–14	7 (9.3)	0 (0.0)	
15–17	44 (58.7)	8 (50.0)	
18–21	24 (32.0)	8 (50.0)	
Center			*0.02*
Egypt	24 (32.0)	10 (62.5)	
Lebanon	51 (68.0)	6 (37.5)	
Anthropometry			
Height, cm (M ± SD)	167.11 ± 7.79	166.93 ± 8.07	0.94
Weight, kg (M ± SD)	116.23 ± 17.85	124.12 ± 20.27	0.12
BMI, kg/m^2^ (M ± SD)	41.48 ± 4.51	44.40 ± 5.11	*0.02*
Metabolic			
Hb, gm/dL (M ± SD)	12.85 ± 1.51	12.6 ± 1.19	0.56
Vit B12, pg/mL (M ± SD)	446.08 ± 180.71	492.7 ± 87.19	0.41
Protein, g/dL (M ± SD)	7.36 ± 0.46	7.31 ± 0.40	0.72
Albumin, g/dL (M ± SD)	4.31 ± 0.32	4.19 ± 0.37	0.29
Ca (mg/dl) (M ± SD)	9.09 ± 0.64	8.50 ± 0.89	*0.046*
HbA1c, % (M ± SD)	5.84 ± 0.91	5.74 ± 1.03	0.74
Associated medical conditions			
Diabetes	14 (18.7)	4 (26.7)	0.49
Hypertension	8 (10.7)	2 (12.5)	0.83
Depression	4 (5.3)	0 (0.0)	0.35
Gastro-esophageal reflux disease	12 (16.0)	2 (12.5)	0.73
Polycystic ovary syndrome	16 (26.7)	4 (30.8)	0.76
Obstructive sleep apnea	15 (20.3)	3 (18.8)	0.89

Cell values represent frequency (percent) unless otherwise stated; *BMI*, body mass index; *LTFU*, lost to follow-up; *m* ± *SD*, mean ± standard deviation; italicized cells indicate statistical significance

This study has limitations. Retrospective analysis of prospectively collected data has limitations regarding causal inference. Our sample size of 91 adolescents, although larger than other OAGB adolescent studies [11, 12], however, an even larger sample size would have been beneficial in enhancing the power of the study. A longer follow-up period of more than 4 years would have provided evidence as to whether the benefits of OAGB were durable and sustained over the long term. A wider range of metabolic and nutritional variables, along with other variables, e.g., socioeconomic factors, medication use, adherence to post-operative care, psychosocial and mental health outcomes, quality of life, behavioral health, or nutritional adherence, would have provided a broader picture of the changes throughout the follow-up after OAGB. All surgical procedures were performed by two experienced surgeons at high-volume centers, which may not reflect outcomes achievable in other settings or by less experienced providers. The study followed up patients for 4 years, and longer-term follow-up to explore any very late nutritional complications or lifelong effects might not have been captured. Most patients were females (81%) and from two Middle Eastern countries, which may limit the applicability of results to male adolescents or other ethnic groups. Appraisal of other commonly affected micronutrients, e.g., iron stores/ferritin, vitamin D, and folate, would have been beneficial. Although our patients who were lost to follow-up (LTFU) were not different compared to those who completed the study across all the variables under examination, however, they tended to have a heavier BMI. Despite this, the current study has many strengths. It is the first to appraise the effectiveness and safety of OAGB at two centers in two countries, employing a larger series of 91 adolescents at four time points after OAGB, appraising a range of anthropometric, nutritional, and metabolic outcomes, as well as the resolution of six obesity-related conditions compared to baseline values.

In summary, OAGB appears to be suitable for adolescents, and although we did not observe micronutrient deficiencies, wider nutritional surveillance is recommended. Future research should address the points highlighted in the limitations, as well as the longer-term impacts on bone health, growth, and development during this critical life stage. In addition, investigations for clear guidance for clinicians about patient selection, optimal timing of intervention, or integration with comprehensive adolescent obesity management would be beneficial, as well as psychosocial aspects, particularly depression, as it deserves special attention and regular objective monitoring dedicated to mental health support. Hence, long-term follow-up is advocated, with practical approaches to maximize compliance in adolescents, including involving the family to optimize outcomes.

## Conclusion

OAGB appears safe and effective in this series of adolescents with obesity. It resulted in significant improvements in anthropometric and metabolic variables, which were maintained for up to 4 years. Although nutritional variables slightly decreased over time, they remained within normal range. OAGB also resulted in early remission of the obesity- associated medical conditions, which were maintained for up to 4 years, except for depression. Early and late complication rates after OAGB for adolescents were very low, with no mortality. These findings suggest that OAGB can be safely recommended for adolescents. Further research beyond 4 years is required to explore whether the benefits are durable in the longer term.

## Data Availability

Data can be shared upon reasonable request and agreement from the institutions where the study was implemented.
